# The Rise of Consumer Health Wearables: Promises and Barriers

**DOI:** 10.1371/journal.pmed.1001953

**Published:** 2016-02-02

**Authors:** Lukasz Piwek, David A. Ellis, Sally Andrews, Adam Joinson

**Affiliations:** 1 School of Management, University of Bath, Bath, United Kingdom; 2 Department of Psychology, Lancaster University, Lancaster, United Kingdom; 3 Division of Psychology, Nottingham Trent University, Nottingham, United Kingdom

## Abstract

Lukasz Piwek and colleagues consider whether wearable technology can become a valuable asset for health care.

**Summary Points**

- Consumer wearables can provide patients with personalized health data, which could assist with self-diagnosis and behaviour change interventions.
- There are a number of concerns about the safety, reliability, and security of using consumer wearables in health care.
- Practitioners and researchers should consider how these technological advances may impact health care in the 21st century.

Will consumer wearable technology ever be adopted or accepted by the medical community? Patients and practitioners regularly use digital technology (e.g., thermometers and glucose monitors) to identify and discuss symptoms. In addition, a third of general practitioners in the United Kingdom report that patients arrive with suggestions for treatment based on online search results [1]. However, consumer health wearables are predicted to become the next “Dr Google.” One in six (15%) consumers in the United States currently uses wearable technology, including smartwatches or fitness bands. While 19 million fitness devices are likely to be sold this year, that number is predicted to grow to 110 million in 2018 [2]. As the line between consumer health wearables and medical devices begins to blur, it is now possible for a single wearable device to monitor a range of medical risk factors (Fig 1). Potentially, these devices could give patients direct access to personal analytics that can contribute to their health, facilitate preventive care, and aid in the management of ongoing illness. However, how this new wearable technology might best serve medicine remains unclear.

**Fig 1:**
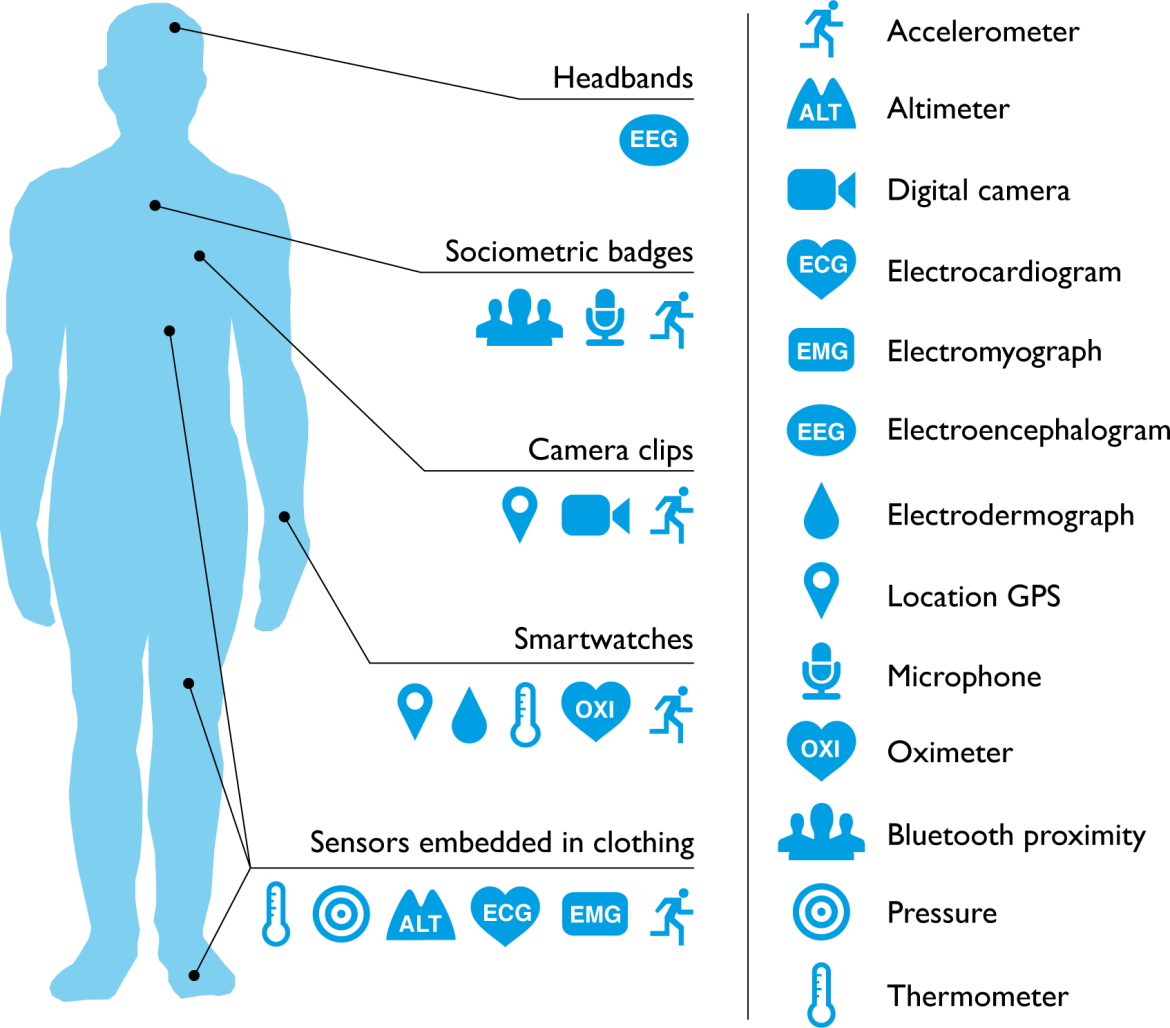
What can consumer wearables do? Heart rate can be measured with an oximeter built into a ring [3], muscle activity with an electromyographic sensor embedded into clothing [4], stress with an electodermal sensor incorporated into a wristband [5], and physical activity or sleep patterns via an accelerometer in a watch [6,7]. In addition, a female’s most fertile period can be identified with detailed body temperature tracking [8], while levels of mental attention can be monitored with a small number of non-gelled electroencephalogram (EEG) electrodes [9]. Levels of social interaction (also known to affect general well-being) can be monitored using proximity detections to others with Bluetooth- or Wi-Fi-enabled devices [10]. Consumer wearables can deliver personalised, immediate, and goal-oriented feedback based on specific tracking data obtained via sensors and provide long lasting functionality without requiring continual recharging. Their small form factor makes them easier to wear continuously. While smartphones are still required to process the incoming data for most consumer wearables, it is conceivable that in the near future all processing functionality will be self contained.

## Do Wearables Affect Behaviour?

### Healthy Individuals

At present, wearables are more likely to be purchased by individuals who already lead a healthy lifestyle and want to quantify their progress [2]. The majority of wearable manufacturers (e.g., Fitbit, Jawbone, and Nike) stress the potential of their devices to become an “all-in-one" platform for improving physical performance and positive habit formation. Wearable manufacturers utilise a range of digital persuasive techniques and social influence strategies to increase user engagement, including the gamification of activity with competitions and challenges, publication of visible feedback on performance utilising social influence principles, or reinforcements in the form of virtual rewards for achievements. There is also a small, but growing, population of wearable users specifically interested in the concept of self-discovery via personal analytics—the Quantified Self (QS) movement [11]. A number of scientific and popular publications describe methods and techniques for using consumer wearables as “self-hacking” devices—to improve sleep, manage stress, or increase productivity [12]. But do these interventions make people healthier?

Current empirical evidence is not supportive. Evidence for the effectiveness of QS methods comes from single-subject reports of users describing their experiences. Subjective reports like these cannot be treated as reliable scientific evidence. Very few longitudinal, randomised controlled studies focus on the impact of wearable technology on healthy users’ behaviour. One exception found that pedometers (and consultations) increased physical activity among older people [13]. It remains unclear how similar interventions may benefit younger adults who are regularly exposed to wearables that provide an ever-increasing stream of behavioural and physiological feedback. Additionally, recent surveys showed that 32% of users stop wearing these devices after six months, and 50% after one year [14]. Many wearables suffer from being a “solution in search of a problem." In other words, they don’t add functional value that is already expected from personal technology of that type, and they require too much effort, which breaks the seamless user experience [15]. Poor implementation of user experience principles [16] alongside the ad hoc design of user interfaces stems in part from the rapid nature of development, which may also explain the lack of randomised trials. Those who market and develop consumer level devices may underestimate the distance between designing a product that appears to be associated with a healthy lifestyle and providing evidence to support this underlying assumption. This is not merely a bureaucratic limitation, as even the best experts are often unable to predict which novel interventions will show benefits when considered as part of a randomised trial [17].

### Patients with a Defined Illness or Comorbidity

How useful are consumer wearables as a patient-driven, “secondary" diagnostic tool? For chronic conditions, wearables could effortlessly provide detailed longitudinal data in order to monitor patients’ progress without involving more sophisticated, uncomfortable, and expensive alternatives. For instance, it is possible to identify the severity of depressive symptoms based on the number of conversations, amount of physical activity, and sleep duration using a wearable wristband and smartphone app [18,19]. Sleep apnoea could be quickly diagnosed, and sleep quality improved, with a lightweight wearable that measures heart rate, breathing volume, and snoring (through tissue vibration) instead of a heavy polysomnograph [20]. Wearables could also feed into a broader system of “predictive preventive diagnosis." For example, a microanalysis of body movement data can be used to detect early symptoms of Parkinson disease [21]. Wearables could provide a platform for at-home management of long-term chronic conditions. Stationary computerised solutions such as web-based services, electronic self-reports, and feedback via emails already facilitate positive behaviour change for such medical issues as obesity [22], anxiety [23], panic disorders [24], post-traumatic stress disorder (PTSD) [25], and asthma [26]. However, those stationary computerised solutions already appear out of date and are almost impossible to use by patients when they are away from their home computer. Despite their widespread use, these solutions result in a high level of patient attrition [27], which might be a result of requiring patients to delay self-report until they are next able to use their home computer [28]. Wearables could address some of the limitations of other interventions by providing instant feedback and offer an individualistic approach while remaining practical [11,15].

In spite of these promises, the actual use of consumer wearables within a clinical population remains limited. The potential applications described above are still in the early stages of development, have not been approved for medical use, and have so far been explored predominantly within an academic research rather than a real-world context. Clinical studies to date that have a closer resemblance to consumer wearables involve (1) pedometers and smartphone apps to tackle a sedentary lifestyle and obesity and (2) home telemonitoring solutions for patients with pulmonary conditions, diabetes, hypertension, and cardiovascular diseases.

The use of pedometers has been associated with significant increases in physical activity and significant decreases in body mass index and blood pressure [29]. Smartphone apps have been shown to complement interventions supporting weight loss [28,30] and increase physical activity [31]. However, interventions involving pedometers and smartphone apps across clinical populations show no evidence of continued behavioural change beyond the duration of the original intervention [29]. There are also inconclusive results regarding home telemonitoring. Reviews illustrating the effects of telemonitoring on clinical outcomes (e.g., a decrease in emergency visits, hospital admissions, and average hospital stay) are more favourable in pulmonary and cardiac patients than in those suffering from diabetes and hypertension [32,33]. However, a number of trials report no beneficial effect of self-monitoring on blood glucose [34], and several demonstrate negative outcomes, including elevated levels of depression [35]. Aspects such as quality of life, acceptability, and cost benefits are infrequently or incompletely reported in telemonitoring trials [33,36], and existing reviews of remote monitoring have frequently been criticised for their poor methodology [37].

## Into the Cloud: Is Wearable-Generated Data Safe, Reliable, and Secured?

This new technology raises additional questions concerning the impact on users’ health and well-being. Currently, wearables exist within a “grey area” regarding user safety. The potential issue of harm is largely absent from the current literature, but it is conceivable that people may become over-reliant on automated systems that provide a false sense of security or fuel a self-driven misdiagnosis [38,39]. Patients could also suffer from negative consequences of excessive self-monitoring by finding it uncomfortable, intrusive, and unpleasant. For instance, several studies have observed that type 2 diabetics who self-monitored their own blood glucose concentration did not benefit from increased glycaemic control but rather found their disease more intrusive [35]. The interaction between a wearable device and a patient is likely to be complex, and further research needs to consider these in more detail. For example, an individual’s personality is likely to play a key role in determining the perceived usefulness of a given device [40].

The reliability and validity of wearable devices is also concerning. Devices are marketed under the premise that they will help improve general health and fitness, but the majority of manufactures provide no empirical evidence to support the effectiveness of their products. Recent comparisons between various wearables for tracking physical activity showed large variations in accuracy between different devices—with error margins of up to 25% [41,42]. This is a serious discrepancy, and it echoes problems witnessed in the medical apps market. For instance, a review in *JAMA Dermatology* showed that smartphone apps for melanoma detection have a 30% failure rate [43]. Lack of reliability is a serious obstacle that needs to be addressed long before a device could be considered for any medical application.

Finally, for patients and medical practitioners, the privacy and security of personal data generated by consumer wearables remains problematic. Users who buy wearable devices today often do not “own” their data. Instead, data may be collected and stored by the manufacturer who sells the device. Being provided with only a summary of results extracted from these data creates a rather odd paradox for the user—they own the device, but not the resulting data. Some manufacturers charge users a monthly fee for access to their own raw data, which is regularly sold to third-party agencies. Other companies are also willing to share a users’ location, age, sex, email, height, weight, or “anonymised” Global Positioning System (GPS)-tracked activities [44,45]. However, “anonymising” data via a simple distortion or removal of identifying features does not provide adequate levels of anonymity and is not sufficient to prevent identity fraud. Sophisticated algorithms can now cross-reference wearable-generated biometric data with other “digital traces” of users’ behaviour. “Digital traces” of behaviour such as time of activity and user location can reveal a person’s identity [46]. Research on “digital traces” from other sources (e.g., social media) demonstrates that these can be alarmingly accurate when it comes to predicting personality [47] and risk-taking behaviours [48], two very individual and personal traits. Furthermore, some wearable devices are easy to hack as a result of various communication technologies that aid the transfer of data between wearables and smartphones [49]. This resonates with similar problems observed in wireless digital pacemakers and glucose pumps, which were vulnerable to cyber-attacks in the past [50,51]. While the consequences of hacks are reduced for noninvasive wearables, a well-coordinated cyber-attack could lead to patient health data being compromised, lost, or distorted.

## Moving Forward: What’s to Come for Wearables in Health Care?

What can make affordable, wearable technology a real asset for health care? One option is to create a simple regulatory framework that doesn’t suppress innovation but helps wearable devices become validated in the context of their health-oriented value. Such an approach was recently discussed in *The New England Journal of Medicine*, but in relation to smartphone health apps’ regulatory status in the US [52]. Authors pointed towards a risk-based classification (e.g., administrative apps, health management apps, and medical apps) that “promotes innovation, protects patient safety, and avoids regulatory duplication" (p. 375 of [52]). As part of this model, the US Food and Drug Administration jurisdiction covers higher-risk medical apps. The National Health Service in the UK adopts a similar pathway with their regulatory framework for mobile apps, which can be classified as “medical devices” by the Medicines and Healthcare Products Regulatory Agency [53]. Applied to a health-oriented wearable device, such a solution could persuade the private sector to provide open access to their data collection practices, analysis methodologies, and measurement concerns. This would address not only the issue of wearables reliability but also secondary concerns relating to data storage and privacy. Apple has recently announced a development of a ResearchKit—an open-source software framework to create smartphone apps and to use wearables for medical research [54]. This is widely perceived as an attempt to accelerate and standardise procedures for regulating Apple’*s* apps alongside wearables as they apply to medical research. We envision that other smartphone and wearable manufacturers will mirror this approach, therefore making it easier for medical researchers to address issues of reliability, safety, and security of patient data. Combining such standardised solutions created by manufacturers with the correct regulatory framework has the potential to accelerate high-quality, large-scale randomised controlled trials in order to deconstruct complex causal interactions and better understand how to make wearables safer and more useful if they are to be adopted in health care.

Another way to address data reliability and behavioural usability issues is to reach the next level in decoding “big data” from wearable devices. Right now, feedback systems built around consumer health wearables are based on simple descriptive statistics—for example, average weekly heart rate and level of activity. Simple summary statistics appear almost trivial given the complex nature of the data that most wearables collect. The same criticism can also be applied to the sociodemographic information recorded by users. The next step will be to move from unsophisticated exploratory feedback to intelligent and personalised explanatory feedback [55]. Interactive computing systems that already exist in smartphones such as Google Now, Apple Siri, and Microsoft Cortana could be used to improve user experience and interaction with wearable technology by making rich data outcomes and feedback more accessible and intelligible [56]. Such systems will be further empowered by the “Internet of Things” (IoT)—a pervasive network of interconnected sensors embedded in everyday spaces and objects that communicate with wearable technology and provide an additional layer of information for users or patients [57]. For example, the Withings system links multiple devices together, including a wearable fitness tracker and sleep sensor placed under the mattress. A smart weight scale also records heart rate, body fat, and air quality, providing even more information about a user’s daily health habits [58]. However, successful applications of “intelligent” computing and the use of multiple consumer sensors requires a truly interdisciplinary approach in order to decode “individual big data.” Computer and data scientists, who write such computational algorithms, have to work closely with clinicians to accurately quantify various health conditions and risk factors. Behavioural scientists and interface designers have to be on board to facilitate and develop more personalised, intuitive, and user-friendly systems of behavioural engagement and feedback. Those whose expertise lies in the design, manufacture, and marketing of consumer wearables should be mindful of the limitations that have plagued previous medical and psychological interventions—specifically, the assumption that the impact of a seemingly positive intervention can be assessed without randomised controlled trials.

## Conclusion

While many champion wearables as data-rich devices that will revolutionise 21st century medicine, it remains highly probable that, like many technological trends, these mass-marketed gadgets will drift into obscurity. However, given their continued popularity, particularly amongst those who already maintain a watchful eye over their lifestyle, health practitioners may need to prepare themselves for an increase in patients who bring wearable data to their next consultation. This may generate additional confusion and anxiety for both practitioner and patient. More worryingly, the margin of error can be high when patients without medical training attempt to attribute symptoms to a specific stream of data from devices that may themselves be unreliable. Drawing a parallel with patient-obtained diagnoses via Google, less than 5% of surveyed health care providers felt that any Internet self-diagnosis was helpful [1]. Alternatively, if frameworks are in place allowing wearable devices to be integrated into health care systems, this could, in turn, kick-start the development of validation programmes that would sit alongside appropriate training for health care professionals. This knowledge and understanding could then be disseminated to patients as validated devices become standardised, providing both individual and aggregated data for patients, governments, and health care providers. Moving forward, practitioners and researchers should try to work together and open a constructive dialogue on how to approach and accommodate these technological advances in a way that ensures wearable technology can become a valuable asset for health care in the 21st century.

## Abbreviations

ECG: electrocardiogram
EEG: electroencephalogram
GPS: Global Positioning System
IoT: Internet of Things
PTSD: post-traumatic stress disorder
QS: Quantified Self
